# Writing the story of immune effector cell‐associated neurotoxicity syndrome

**DOI:** 10.1002/jha2.843

**Published:** 2024-01-03

**Authors:** Vincent Bonny, Tomas Urbina, Antoine Capes, Jeremie Joffre

**Affiliations:** ^1^ Service de Médecine Intensive Réanimation Hôpital Saint‐Antoine Assistance Publique ‐ Hôpitaux de Paris Paris Cedex 12 France; ^2^ Sorbonne Université Paris France; ^3^ Service d'hématologie et thérapie cellulaire Hôpital Saint‐Antoine Assistance Publique ‐ Hôpitaux de Paris Paris Cedex 12 France

**Keywords:** cell toxicity, cellular therapies, chimer

## 1 INTRODUCTION

A 48‐year‐old patient received chimeric antigen receptor (CAR)‐T cell therapy for relapsed diffuse large‐B cell lymphoma with central nervous system involvement. He had no sign of active disease at the time of CAR‐T cell infusion, after being treated with Rituximab, Ifosfamide, Carboplatine, Etoposide (R‐ICE) for left capsulolenticular and temporal lesions responsible for right‐facial paralysis.

Two days after CAR‐T cell infusion (Axicabtagene‐ciloleucel), the patient became febrile and four days after infusion he received Tocilizumab given that persistent fever.

**FIGURE 1:**
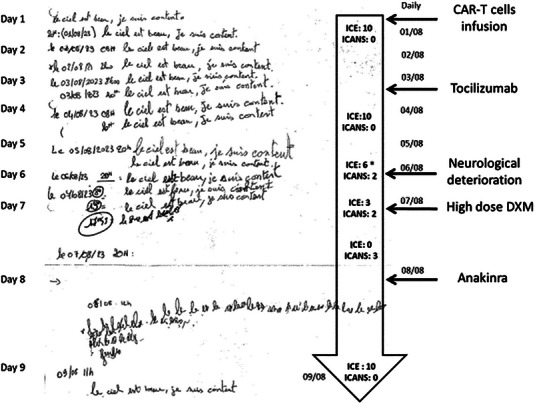
Picture showing results of twice‐daily patient's ICE‐score writing section over time after CAR‐T cells infusion. The sentence chosen is “le ciel est beau, je suis content”, meaning in French “The sky is beautiful, I'm happy”. ICE: Immune Effector Cell‐associated Encephalopathy; DXM: Dexamethasone. **ICE score (10 points scale)**. ‐Orientation: Orientation to year, month, city, and hospital: 4 points (1 point each). ‐Naming: Name 3 objects (e.g., clock, pen, and button): 3 points (1 point each). ‐Following commands: (e.g., Show me 2 fingers or close your eyes and stick out your tongue): 1 point. ‐Writing: Ability to write a standard sentence (e.g., Our national bird is the bald eagle): 1 point. ‐Attention: Count backward from 100 by 10: 1 point. *ICE Score 6 legend: 2 points missing on orientation, 1 point missing on naming, and 1 point missing on attention (unable to count backward).

The daily dysgraphia evaluation as well as immune effector cell‐associated encephalopathy (ICE) Score and neurological exam had been normal until day 6, when slight difficulties in writing the usual sentence appeared (Figure 1). This progressive dysgraphia was followed by more overt neurological deterioration with confusion, aphasia, and diffuse hyperreflexia. ICE‐score decreased from 10 to 6, Immune effector cell‐associated neurotoxicity syndrome (ICANS) grade 2 was suspected. On day 7, the patient could not write and the ICE score was 0 with grade 3 ICANS. Echolalia with apraxia appeared and aphasia worsened. Electroencephalogram revealed encephalopathy and brain magnetic resonance imaging ruled out encephalitis, edema, bleeding, or lymphoma progression. Cerebrospinal fluid examination revealed 6 leukocytes/mm3 and elevated proteinorachia (3.39 g/L).

The patient received high‐dose Dexamethasone (10 mg every 6 h) plus Anakinra (200 mg every 8 h) and, on the following day, could only write a single word repeatedly. 48 h after the treatment he could normally write the complete sentence again and the ICE score was 10.

While paraclinical exams might help to manage treatment response in complex patients, clinicians should stay aware that early diagnosis and follow‐up can rely on simple clinical findings such as handwriting.

## AUTHOR CONTRIBUTIONS

All authors took care of the patient. Vincent Bonny wrote the first draft of the manuscript. All authors read and approved the final manuscript.

## CONFLICT OF INTEREST STATEMENT

The authors declare no conflict of interest.

## ETHICS STATEMENT

The authors have confirmed ethical approval statement is not needed for this submission.

## PATIENT CONSENT STATEMENT

The authors have confirmed patient consent statement is not needed for this submission.

## CLINICAL TRIAL REGISTRATION

The authors have confirmed clinical trial registration is not needed for this submission.

## Data Availability

The data that support the findings of this study are available on request from the corresponding author.

